# Granuloma annulare following acetazolamide challenge/rechallenge in a patient with idiopathic intracranial hypertension

**DOI:** 10.1016/j.jdcr.2024.12.015

**Published:** 2024-12-26

**Authors:** Samantha A. Mannala, Lulu L.C.D. Bursztyn

**Affiliations:** a College of Medicine, University of Saskatchewan, Saskatoon, Saskatchewan, Canada; b Department of Ophthalmology, Western University, London, Ontario, Canada; c Department of Clinical Neurological Sciences, Western University, London, Ontario, Canada

**Keywords:** acetazolamide, granuloma annulare, idiopathic intracranial hypertension

Acetazolamide, a sulfonamide derivative, and direct carbonic anhydrase inhibitor, has Food and Drug Administration-approved indications for idiopathic intracranial hypertension (IIH), glaucoma, periodic paralysis, epilepsy, congestive heart failure, and altitude sickness. Acetazolamide has a broad range of side effects, which include fatigue, nausea, vomiting, abdominal pain, paresthesia, and metallic taste. Rarer side effects include Stevens-Johnson syndrome and toxic epidermal necrolysis.^1^

Granuloma annulare (GA) is a rare benign skin condition that appears as skin-colored to erythematous papules and plaques. The etiopathogenesis is unknown, but several causative medications have been identified, including allopurinol, amlodipine, anti–tumor necrosis factor-alfa agents, immunizations, botulinum toxin, and topiramate. Rarely, acetazolamide, apremilast, ixekizumab, and phototherapy have also been implicated in inducing GA.^2^ We report a rare case of biopsy-proven GA after challenge/rechallenge with acetazolamide occurring in a patient with IIH.

A 56-year-old woman was diagnosed with IIH and prescribed acetazolamide 750 mg daily. She was on no other medications at the time of presentation and her only other medical history was diverticulitis. She had been on acetazolamide for 2 years when she presented with a rash on her hands and legs that she described as itchy and sore (Fig 1). The rash was eventually biopsied and demonstrated ill-defined palisading granuloma and perivascular lymphocytic infiltrate, compatible with GA. At this time, the IIH was well controlled, with best-corrected visual acuity of 20/20 in both eyes and resolution of both headaches and papilledema. She was then tapered off acetazolamide and the rash faded over months.

**Fig 1:**
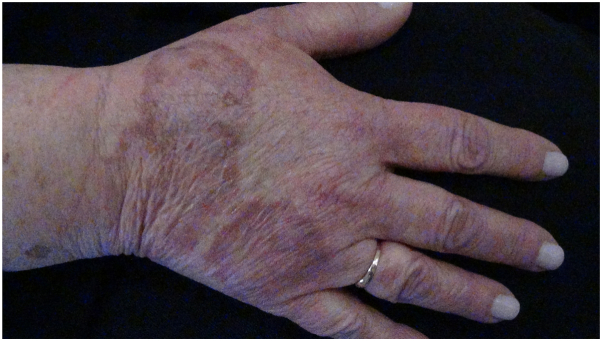
Erythematous annular rash on the hand, which was biopsied and confirmed to be granuloma annulare.

Approximately 1 year later, she had recurrence of headaches and papilledema with corresponding increase in retinal nerve fiber layer on optical coherence tomography. Due to the recurrence of IIH, acetazolamide was restarted at 1 g daily.

When she returned 3 months later, her headaches had improved on acetazolamide, but she had another episode of a rash on her foot that was clinically consistent with GA (Fig 2). Blood work, including complete blood count, C-reactive protein, antinuclear antibody, and antiphospholipid antibodies were all normal. The option of switching to a different medication was offered, but the patient elected to stay on acetazolamide despite ongoing rash, because of her well controlled symptoms.

**Fig 2:**
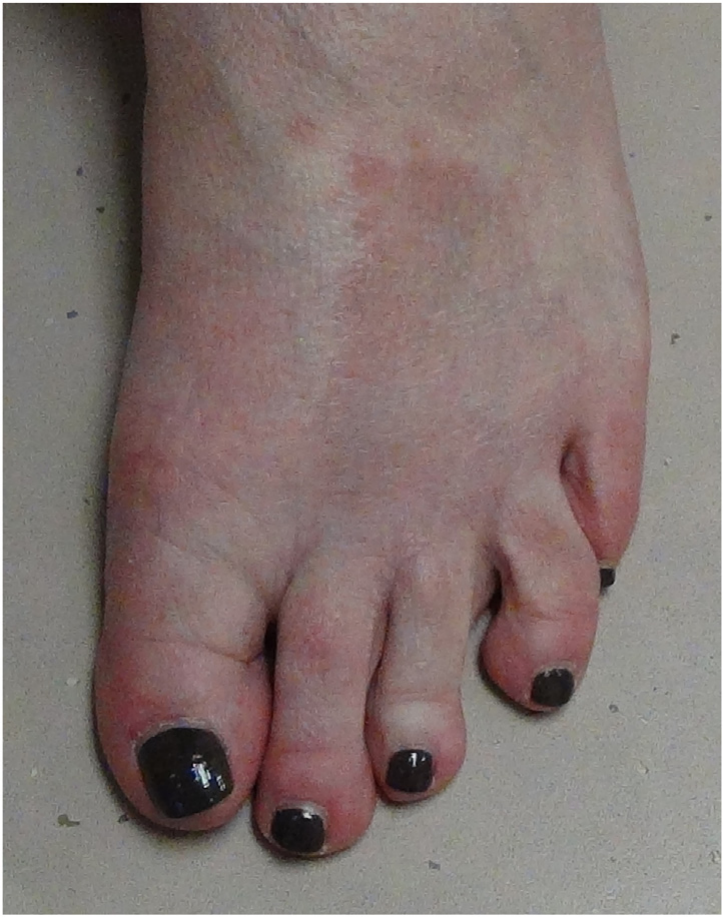
Erythematous annular rash on the foot, clinically consistent with granuloma annulare. This rash appeared after the patient restarted acetazolamide.

To our knowledge, this is the first reported case of papular/plaque type GA induced by acetazolamide and the second reported case of acetazolamide-induced GA overall. The other published case was subcutaneous GA that occurred in a pediatric patient, also receiving acetazolamide for treatment of IIH.^3^ Topiramate, like acetazolamide, is a sulfonamide derivative and carbonic anhydrase inhibitor and has a more frequently reported association with GA.^4^^,^^5^ The precise mechanism by which topiramate may cause GA has not been established but may be secondary to a delayed-type hypersensitivity reaction.^4^ Prior studies have implicated overactivation of the T helper 1 pathway in GA, which supports a delayed-type hypersensitivity reaction induced by the T helper 1 pathway.^2^ Due to the similarities in drug class between acetazolamide and topiramate, we hypothesize that acetazolamide may induce GA through a similar mechanism. Acetazolamide-induced GA fits into a broader category of immune-mediated reactions called interstitial granulomatous drug reaction, a term used to describe a rare cutaneous condition, typically appearing as an erythematous to violaceous annular plaque.^6^ Interstitial granulomatous drug reaction is characterized by granulomatous inflammation arising in response to a drug, with histopathologic features of interstitial granulomas and neutrophilic infiltration. Furosemide, another sulfa-containing drug, has been linked to this type of immune response, including delayed-type hypersensitivity, which is often involved in granulomatous reactions.^6^

A rare feature of this case is the challenge/rechallenge phenomenon, with improvement of GA after discontinuation of acetazolamide and recurrence after restarting the drug. We believe a strong causal relationship is established given that, (1) the GA appeared after initiating acetazolamide, (2) the GA improved after discontinuation of acetazolamide, (3) new lesions appeared after restarting acetazolamide, (4) the lack of other medications or medical conditions associated with GA, and (5) normal laboratory testing for autoimmune disease.

## Conflicts of interest

None disclosed.
